# A CNN-MAMBA-Based Framework for Salient Bowel Sound Detection and Gastrointestinal Health Assessment

**DOI:** 10.3390/s26123768

**Published:** 2026-06-12

**Authors:** Zixuan Zeng, Lijing Yang, Chen Zhou, Ling He, Junyi Yang, Hong Mao, Jing Zhang

**Affiliations:** 1 College of Biomedical Engineering, Sichuan University, Chengdu 610065, China; 2023141670101@stu.scu.edu.cn (Z.Z.); 2023141670103@stu.scu.edu.cn (L.Y.); 2023141670124@stu.scu.edu.cn (C.Z.); jing_zhang@scu.edu.cn (J.Z.); 2 Department of Anorectal Surgery, The Sichuan Second Hospital of Traditional Chinese Medicine, Chengdu 610031, China; maohong@sc2zyy.com

**Keywords:** bowel sound analysis, salient event detection, CNN-MAMBA, CNN-Conformer-MIL, multi-view spectral representation, gastrointestinal health assessment, constipation classification, elderly population

## Abstract

With the rapid aging of the global population, constipation has become a major gastrointestinal concern among elderly individuals. Bowel sounds provide a non-invasive acoustic signal for assessing gastrointestinal function, but their automatic analysis remains challenging due to sparsity and non-stationarity. This study proposes a two-stage bowel sound analysis framework based on continuous abdominal recordings. First, a Convolutional Neural Network-MAMBA (CNN-MAMBA) model was used for salient bowel sound detection. Second, a patient-level constipation classification model was developed using multi-view spectral representations and a Convolutional Neural Network-Conformer-Multiple Instance Learning (CNN-Conformer-MIL) architecture. On a held-out test set, the detection model achieved an accuracy of 0.87, an F1-score of 0.78, and a ROC-AUC of 0.93. For patient-level classification under binary Bristol Stool Form Scale (BSFS) grouping, five-fold cross-validation yielded a mean accuracy of 0.665 and an F1-score of 0.755. All BSFS labels were annotated by clinical physicians and temporally aligned with bowel sound recording. Given the modest improvement and cross-validation variability, the patient-level results should be interpreted as preliminary feasibility evidence. These findings suggest that bowel sound analysis may serve as an auxiliary screening or longitudinal monitoring tool rather than a stand-alone diagnostic system.

## 1. Introduction

With the rapid aging of the global population, gastrointestinal dysfunction has become an important health concern among older adults [1]. Chronic constipation is common in elderly populations and is often associated with reduced intestinal motility, chronic diseases, medication use, and age-related physiological changes [2,3,4,5]. It can impair quality of life and increase healthcare burdens, highlighting the need for objective and non-invasive assessment methods.

Bowel sounds are generated by gastrointestinal motility and gas–fluid interactions, and have been explored as a non-invasive acoustic signal for bowel function assessment [6]. Compared with invasive examinations, abdominal acoustic monitoring is low-cost, convenient, and suitable for continuous observation [7,8]. However, bowel sounds are usually sparse, short-duration, and non-stationary, arising from intestinal smooth-muscle contractions and the movement of luminal contents. Their acoustic patterns may be influenced by gastrointestinal motility and can vary with systemic or behavioral factors, such as blood pressure, heart rate, and recent physical activity, making automatic detection and analysis challenging.

Previous studies have investigated bowel sound detection, segmentation, and recognition using signal processing and machine learning methods. Zhang et al. [9] proposed a defecation prediction model based on iterative kurtosis detection, Kalman filtering, handcrafted time–frequency features, and a BP neural network. Their study used 232 bowel sound records from six subjects and achieved an average prediction accuracy of 88.71%. This work showed the feasibility of bowel sound-based prediction, but the task focused on defecation intention rather than constipation-related assessment.

Deep learning methods have further improved automated bowel sound analysis. Sitaula et al. combined a CNN with a Laplace Hidden Semi-Markov Model for neonatal bowel sound detection, showing that temporal modeling can improve event detection stability [10]. Baronetto et al. studied multiscale bowel sound event spotting in highly imbalanced wearable monitoring data [11], while Zhang et al. developed a lightweight deep learning-based bowel sound segmentation algorithm using convolutional and bidirectional GRU layers [12]. Yu et al. introduced self-attention and self-supervised pre-training for bowel sound recognition [13]. Kalahasty et al. further proposed a You Only Listen Once (YOLO) deep learning model for automatic prominent bowel sound detection in healthy subjects [14], while Mansour et al. benchmarked different machine learning strategies for bowel sound pattern classification [15]. These studies demonstrate the progress of automated bowel sound analysis, but most still focus on signal-level detection, segmentation, or recognition.

More recently, Baronetto et al. extended bowel sound event spotting to a wearable monitoring scenario for inflammatory bowel disease detection, combining automated bowel sound retrieval, feature extraction, and gradient boosting classification, while also analyzing classification window size, feature relevance, and the association between bowel sound-based classification and disease activity [16]. This study is particularly relevant to the present work because it highlights the importance of benchmarked model selection and feature-level interpretability when translating bowel sound analysis from event detection to patient-level health assessment. Beyond bowel sounds, recent biomedical AI literature has emphasized that model selection should be guided by task characteristics, data scale, comparison with appropriate baselines, calibration, and interpretability rather than model complexity alone [17]. These considerations motivated our use of multiple baseline comparisons, ablation analyses, patient-level calibration, and cautious interpretation of feasibility-oriented classification results.

The BSFS is a standardized tool for stool form classification and is closely related to clinical evaluation of bowel function [18,19]. Stool form has also been shown to correlate with intestinal transit time and gut microbiota characteristics [20,21]. Therefore, BSFS provides a clinically meaningful reference for exploring the relationship between bowel sound patterns and constipation-related status.

Despite recent progress, the relationship between automatically detected bowel sound events and BSFS-based bowel status remains insufficiently explored, especially in elderly populations. To address this gap, this study proposes a two-stage bowel sound analysis framework for elderly subjects. The framework first identifies salient bowel sound events from continuous abdominal recordings using a CNN-MAMBA model and then explores whether the extracted bowel sound information can support constipation-related assessment based on BSFS grouping. This study aims to evaluate the feasibility of using bowel sounds as a non-invasive auxiliary signal for gastrointestinal health assessment in elderly populations.

## 2. Methods

### 2.1. Problem Formulation

Bowel sound analysis aims to extract physiologically meaningful information from continuous abdominal acoustic recordings. In clinical practice, manual annotation of bowel sounds is time-consuming, labor-intensive, and often subject to inter-observer variability, which limits its scalability and reliability.

To address these challenges, we formulate bowel sound analysis as a two-stage learning problem, consisting of salient bowel sound detection and subsequent patient-level health classification. This design mimics the clinical workflow, where relevant bowel sound events are first identified before further diagnostic analysis.

(1) Salient Bowel Sound Detection

Given a continuous abdominal audio signal of the *n*-th patient, denoted as $x_{n}\left(t\right)$, the first objective is to identify acoustically meaningful bowel sound events.

After preprocessing and segmentation, the signal is decomposed into a set of candidate segments:

(1) $$B_{n}={\left\{b_{n,j}\right\}}_{j=1}^{m_{n}}$$

where $b_{n,j}$ represents the *j*-th segment and $m_{n}$ is the total number of segments for patient n.

The detection task is formulated as a binary classification problem:

(2) $$y_{n,j}=f_{\det}(b_{n,j})$$

where $y_{n,j}\in \{0,1\}$ indicates whether the segment corresponds to a salient bowel sound event.

(2) Patient-Level Bowel Sound Classification

Given the segment set $B_{n}$, the second objective is to infer the patient-level bowel condition based on multiple segment-level observations.

For each segment, a prediction probability is obtained:

(3) $$p_{n,j}=f_{cls}(b_{n,j})$$

where $f_{cls}(\cdot )$ denotes the segment-level classification model.

The patient-level prediction is obtained by aggregating segment-level outputs:

(4) $$p_{n}=G(\left\{p_{n,j}\right\})=G({\left\{p_{n,j}\right\}}_{j=1}^{m_{n}})$$

where G(·) denotes an aggregation function over the set of segment-level predictions.

To further enhance prediction reliability, a calibration function is applied:

(5) $${\tilde{p}}_{n}=H(p_{n})$$

where H(·) represents a learnable calibration mapping that refines the aggregated prediction.

Finally, the predicted label is determined as

(6) $${\hat{y}}_{n}=I({\tilde{p}}_{n}>\tau )$$

where τ is the decision threshold.

(3) Overall Formulation

The entire framework can be summarized as a hierarchical mapping:

(7) $${\tilde{p}}_{n}=H(G(\{f_{\mathrm{cls}}(b_{n,j})|b_{n,j}\in f_{\det}(x_{n}(t))\}))$$

which integrates segment-level acoustic modeling and patient-level decision making into a unified formulation.

### 2.2. Overview of the Proposed Framework

**Figure 1:**
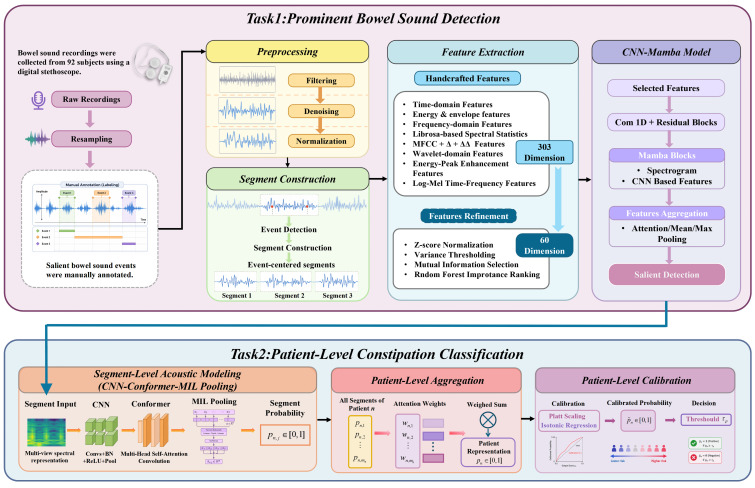
Overall framework of the proposed bowel sound analysis system. The framework includes five main stages: (1) preprocessing, (2) segment construction, (3) feature extraction and fusion, (4) salient segment detection using a CNN-MAMBA model, and (5) constipation classification from segment level to patient level.

As illustrated in Figure 1, the proposed framework provides an end-to-end pipeline to sequentially perform salient bowel sound detection and patient-level health classification.

Bowel sound signals exhibit inherent characteristics of sparsity, intermittency, and non-stationarity. In continuous recordings, informative bowel sound events typically appear as short-duration bursts embedded within long periods of background activity. Directly modeling the entire signal may introduce substantial redundancy and noise, which can degrade model performance. To address this issue, the proposed framework first converts continuous recordings into a set of candidate acoustic segments through preprocessing and segment construction, thereby transforming the problem into a more tractable segment-level analysis task.

In terms of feature representation, considering that bowel sound signals contain complementary information across spectral structure, energy distribution, and temporal dynamics, task-specific representation strategies are adopted. For salient bowel sound detection, both statistical acoustic descriptors and time-frequency representations are extracted as handcrafted feature modalities. These features are not treated as independent predictive inputs but are instead organized into structured representations and fed into a deep learning model, enabling the network to learn higher-level abstractions while preserving domain-specific prior information. For patient-level constipation classification, a multi-view spectral representation is introduced to capture diverse acoustic characteristics from different perspectives, thereby enhancing both discriminative capability and robustness.

The overall framework consists of two main stages.

In the first stage, salient bowel sound detection is performed at the segment level. Each candidate segment is processed by a CNN-MAMBA model, which combines convolutional structures for local feature extraction with state-space modeling for long-range dependency learning. This stage identifies acoustically meaningful bowel sound events and filters out irrelevant or low-information segments, effectively improving the signal-to-noise ratio for subsequent analysis.

In the second stage, patient-level constipation classification is conducted based on the selected segments. The retained segments are represented using multi-view spectral features and fed into a CNN-Conformer-MIL model to obtain segment-level predictions. The Conformer module captures both global temporal dependencies and local contextual continuity, while the MIL mechanism accounts for the varying contributions of different segments to the final decision. The segment-level outputs are then aggregated and further refined through a patient-level calibration module, which models the distribution of predictions to produce a more stable and reliable patient-level outcome.

Overall, the proposed framework is designed in a task-driven and signal-aware manner. By integrating event-driven segmentation, multi-view representation learning, deep acoustic modeling, and distribution-aware patient-level decision making, the framework progressively enhances the discriminative capability and robustness of bowel sound analysis across multiple levels.

### 2.3. Task 1: Salient Bowel Sound Detection

For salient bowel sound detection, continuous recordings were decomposed into 0.5 s candidate segments with a 0.1 s hop and represented by refined handcrafted acoustic features before CNN-MAMBA classification.

#### 2.3.1. Preprocessing and Candidate Segment Construction

To improve signal consistency and reduce the influence of background interference, the raw abdominal audio recordings were first subjected to a preprocessing pipeline. Specifically, all recordings were resampled to 4000 Hz to ensure a unified sampling condition across subjects and to preserve sufficient temporal resolution for capturing bowel sound dynamics.

Bowel sounds are generated by gastrointestinal motility and fluid-gas interactions within the intestine, and their dominant spectral components are typically distributed within a mid-frequency range. Based on this physiological characteristic, a bandpass filter with a passband of 80–1800 Hz was applied to suppress low-frequency baseline drift and high-frequency noise while preserving the most informative acoustic components related to bowel activity.

After filtering, amplitude normalization was performed to reduce inter-recording variability caused by differences in sensor placement, abdominal contact conditions, and individual physiological differences. This step ensures that the model focuses on relative acoustic patterns rather than absolute amplitude variations.

For salient bowel sound detection, candidate segments were generated from each continuous recording using a sliding-window strategy. Given a preprocessed signal $x_{n}(t)$, the j-th candidate segment can be written as

(8) $$b_{n,j}\;=x_{n}(t),\;\;t\in [t_{j},t_{j}+w]$$

where w denotes the window length and $t_{j}$ is the start position of the segment. In this study, the window length and hop size were set to 0.5 s and 0.1 s, respectively, resulting in overlapping segments that provide dense temporal coverage of transient bowel sound events.

To assign labels to each candidate segment, an overlap-based rule was employed according to its temporal relationship with manually annotated bowel sound intervals. Let $e_{r}$ denote an annotated event. The overlap ratio between segment $b_{n,j}$ and event $e_{r}$ is defined as

(9) $$\mathrm{lo}O(b_{n,j},e_{r})=\frac{|b_{n,j}\cap e_{r}|\;}{|b_{n,j}|}$$

where $\left|b_{n,j}\cap e_{r}\right|$ is the intersection duration between the segment and the annotated event, and $\left|b_{n,j}\right|$ is the segment duration. In this study, a segment was labeled as positive when its maximum overlap ratio with any annotated bowel sound interval was greater than or equal to 0.30. A segment was labeled as negative when its maximum overlap ratio was less than or equal to 0.05. Segments with overlap ratios between 0.05 and 0.30 were treated as gray-zone samples and were excluded from model training to reduce boundary ambiguity and label noise.

For each annotated event, event-centered candidate windows were additionally generated around the annotation midpoint, and hard-negative samples were retained from high-energy non-event regions. This sampling strategy was used only within the training portion of each split, while validation and test segments were generated by the same fixed sliding-window and overlap-labeling rules.

#### 2.3.2. Feature Representation

Bowel sounds are short-duration, non-stationary acoustic signals generated by gastrointestinal motility and the interaction among intestinal contents, gas flow, and peristaltic movement. Such physiological processes lead to complex temporal fluctuations, energy variations, and nonlinear acoustic patterns. Therefore, extracting informative and robust acoustic features is essential for effective bowel sound modeling.

In this study, handcrafted acoustic descriptors were adopted to provide structured and interpretable representations of bowel sound signals. These features capture complementary information from multiple perspectives and serve as the input to the subsequent deep learning model.

Handcrafted Features

Handcrafted features were extracted to characterize bowel sounds from multiple complementary perspectives, including temporal morphology, energy variation, envelope fluctuation, spectral distribution, cepstral structure, wavelet-domain properties, and time–frequency patterns. According to the implemented feature extraction pipeline, the initial feature representation consisted of 303 dimensions in total. This representation included conventional handcrafted acoustic descriptors and a 64-dimensional Log-Mel time–frequency feature vector.

Specifically, time-domain features were first extracted to describe the basic waveform morphology of each bowel sound segment. These features included the mean, standard deviation, variance, root mean square value, peak amplitude, peak-to-peak amplitude, median, interquartile range, skewness, kurtosis, zero-crossing rate, shape factor, crest factor, impulse factor, margin factor, and clearance factor. These descriptors reflect the overall amplitude distribution, waveform fluctuation, and impulsive characteristics of bowel sounds.

Energy- and envelope-related features were then extracted to characterize the short-term intensity variation in bowel sounds. For frame-level energy analysis, the frame length and hop length were set to 128 and 64 samples, respectively. Short-time energy and frame-level RMS were calculated for each frame, and statistical summaries including the mean, standard deviation, minimum, maximum, and median were computed. In addition, the Hilbert transform was used to obtain the amplitude envelope of each segment, from which envelope statistics, peak amplitude, peak-to-mean ratio, and standard-deviation-to-mean ratio were further derived. These features were designed to capture the burst-like and intermittent nature of bowel sound events.

Frequency-domain features were extracted using the magnitude spectrum of each segment. The extracted descriptors included dominant frequency, spectral centroid, spectral bandwidth, spectral entropy, spectral roll-off at 85% and 95% energy levels, spectral flatness, and band energy ratios across several frequency ranges, including 0–200 Hz, 200–400 Hz, 400–800 Hz, 800–1200 Hz, 1200–1800 Hz, and above 1800 Hz. These features describe the distribution of acoustic energy across frequency bands and help distinguish bowel sounds from background noise and other physiological acoustic interference.

In addition to manually calculated frequency-domain descriptors, librosa-based spectral statistics were also extracted, including spectral centroid, spectral bandwidth, spectral roll-off, spectral flatness, and zero-crossing rate. For each of these frame-level descriptors, statistical functionals such as mean, standard deviation, minimum, maximum, and median were computed to obtain a fixed-length representation.

Cepstral features were extracted using Mel-frequency cepstral coefficients. In this study, 13 MFCC coefficients were computed for each segment. For each coefficient, the mean, standard deviation, minimum, and maximum were calculated. To further describe dynamic acoustic changes, first-order delta MFCC and second-order delta-delta MFCC features were also extracted, with the mean and standard deviation calculated for each coefficient. These features provide a compact representation of perceptual acoustic characteristics and temporal spectral variation.

Wavelet-domain features were further extracted to capture transient and non-stationary properties of bowel sounds. The db4 mother wavelet was used with a decomposition level of 4. For each wavelet coefficient group, the mean, standard deviation, energy, and energy ratio were calculated, and wavelet entropy was additionally computed to describe the distribution of signal energy across decomposition levels.

Furthermore, energy-peak enhancement features were designed to emphasize prominent acoustic events associated with bowel activity. These features included generalized frame energy, smoothed envelope statistics, top-k envelope amplitude statistics, smoothed short-time energy statistics, envelope peak count, peak density, peak amplitude statistics, peak prominence statistics, and peak interval statistics. This design aims to strengthen the representation of short-duration burst patterns, which are typical characteristics of bowel sound events.

Finally, Log-Mel time-frequency features were extracted to provide a structured spectral representation of each segment. The Log-Mel spectrogram was normalized and fixed to a consistent temporal length. A 64-dimensional time-frequency feature vector was obtained by averaging the normalized Log-Mel representation along the temporal axis. This vector was included in the initial 303-dimensional feature representation, while the full Log-Mel sequence was also saved separately for pure deep-learning input.

Among the handcrafted descriptors, short-time energy is defined as

(10) $$E=\sum_{i=1}^{N}x_{i}^{2}$$

where $x_{i}$ denotes the bowel sound segment and N is the number of samples. In addition, the spectral centroid was used to describe the center of spectral energy distribution:

(11) $$C=\frac{\sum_{f}f\cdot |X(f)|}{\sum_{f}|X(f)|}$$

where X(f) is the spectral magnitude at frequency f. The spectral bandwidth was further used to describe spectral dispersion. These handcrafted features preserve interpretable acoustic characteristics and provide explicit descriptors associated with bowel sound intensity, temporal variation, and frequency composition.

Feature Refinement

To reduce redundancy and improve representation compactness, a feature refinement strategy was applied to the handcrafted features. First, z-score normalization was performed to standardize feature scales and reduce the influence of feature magnitude differences. Second, low-variance features were removed through variance thresholding to eliminate features with limited discriminative information. Third, mutual information was used to retain the top informative dimensions, followed by random forest importance ranking for further refinement.

Through this process, the original 303-dimensional fused feature vector was reduced to a compact 60-dimensional representation. Therefore, although the initial feature extraction stage produced 303-dimensional features, the final input to the CNN-MAMBA model was the refined 60-dimensional feature vector.

#### 2.3.3. CNN-MAMBA-Based Detection Model

**Figure 2:**
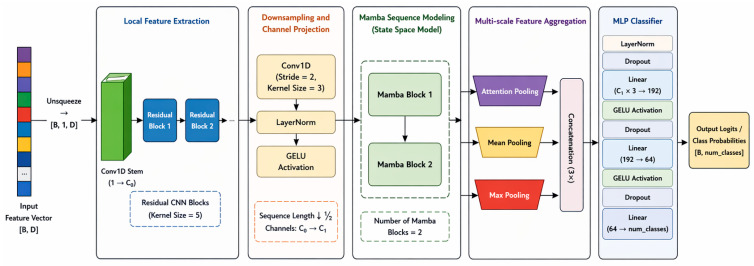
Architecture of the proposed CNN-MAMBA model for salient bowel sound detection.

The overall architecture of the proposed CNN-MAMBA model is illustrated in Figure 2. The model is designed to identify salient bowel sound events from candidate segments by integrating local feature modeling and global dependency modeling within a unified framework.

Each input sample is represented by the fused feature vector obtained in Section 2.3.2. Let the input feature be denoted as z, and the batch input as Z. For convolutional processing, the input is reshaped into a one-dimensional feature sequence.

The proposed CNN-MAMBA model consists of three main stages: local feature extraction, global sequence modeling, and multi-scale feature aggregation, followed by a classification head.

In the first stage, a convolutional stem followed by residual convolutional blocks is employed to extract local feature interactions within the fused feature space [22]. The convolutional transformation can be expressed as

(12) $$h^{\left(c\right)}=\phi (\mathrm{Conv}(z))$$

where Conv(·) denotes one-dimensional convolution and ϕ(·) represents a nonlinear activation function. The residual structure enhances feature propagation and enables the model to capture interactions among heterogeneous feature components.

Before entering the sequence modeling stage, the intermediate representation is downsampled using a stride-2 convolutional layer and projected to a higher channel dimension. This operation reduces the sequence length while enriching channel-wise representations, thereby improving computational efficiency.

In the second stage, a MAMBA-based sequence modeling module is introduced to capture long-range dependencies across the feature sequence [23]. Let the intermediate sequence be denoted as $h^{\left(s\right)}$, then the context-aware representation is obtained as

(13) $$h^{\left(m\right)}=Mamba(h^{\left(s\right)})$$

The MAMBA module, based on state-space modeling, enables efficient modeling of long-range dependencies and temporal dynamics, which are essential for representing non-stationary bowel sound patterns.

In the third stage, multi-scale feature aggregation is performed to summarize the sequence representation. Specifically, attention pooling, mean pooling, and max pooling are applied in parallel, and their outputs are concatenated as

(14) $$h\;=[h_{att},h_{mean}\;,\ldots ,h_{\max}\}$$

This aggregation strategy integrates complementary statistical perspectives and enhances representation robustness.

Finally, the aggregated feature vector is fed into a multilayer perceptron classifier to produce the output logits:

(15) o  =MLP(h)

where o denotes the predicted logits for the input segment, which can be transformed into class probabilities via a sigmoid function. In this study, the model is used for salient bowel sound detection at the segment level.

### 2.4. Task 2: Patient-Level Constipation Classification

For patient-level constipation classification, the detected salient segments from each subject were converted into multi-view spectral representations, processed by the CNN-Conformer-MIL model, and then aggregated into a patient-level prediction.

#### 2.4.1. Segment Construction Based on Salient Bowel Sound Events

For each subject, the candidate pool for patient-level classification was constructed from automatically detected salient bowel sound events. Specifically, each detected event was used to define an acoustically active segment, which was more likely to contain classification-relevant information than long periods of background or low-activity signals. This event-driven segmentation strategy allowed the model to focus on potentially informative bowel sound patterns while reducing the influence of background noise.

To control computational cost and maintain a comparable input scale across subjects, an upper limit was applied to the number of retained candidate segments. When more than 64 candidate segments were available for a subject, the 64 segments with the highest detection confidence were retained; otherwise, all detected segments were used.

The resulting segments were treated as independent acoustic units and further processed by the feature extraction and modeling modules described below.

#### 2.4.2. Multi-View Spectral Representation

To effectively characterize bowel sound signals, a multi-view spectral representation is constructed as the input feature at the segment level. This design serves as an important component of the proposed framework by integrating complementary acoustic information beyond conventional single-view spectral representations.

Given an input bowel sound segment x(t), its time-frequency representation is first obtained via short-time Fourier transform:

(16) X(f,t)=STFT(x(t))

Based on this representation, three complementary spectral views are constructed. First, the log-Mel [24] spectrogram is computed using 64 Mel filter banks to capture the global time-frequency structure:

(17) $$S_{mel}(f,t)=\log(Mel(|X(f,t){|}^{2}))$$

This results in a time–frequency representation of size 64×T, where T denotes the number of temporal frames. To ensure compatibility with the downstream neural network, the spectrogram is further resized to a fixed resolution of 32×32. This representation provides a perceptually meaningful description of spectral energy distribution.

Second, the PCEN representation is introduced to enhance weak but informative acoustic components and suppress background variations [25]. Unlike conventional logarithmic compression, PCEN performs adaptive gain control and dynamic normalization, making it more robust to non-stationary noise. This mechanism is particularly beneficial for bowel sound signals, which often contain low-energy bursts embedded in background-dominated recordings.

Third, temporal difference features are incorporated to model local dynamic changes along the time axis:

(18) $$\Delta S(f,t)=S_{mel}(f,t)-S_{mel}(f,t-1)$$

which highlight transient acoustic patterns and temporal variations, and are especially effective in capturing the burst-like and intermittent characteristics of bowel sounds.

These complementary components are integrated through weighted fusion to form a unified multi-view representation:

(19) $$S_{mv}=\alpha_{1}S_{mel}+\alpha_{2}S_{pcen}+\alpha_{3}\Delta S$$

where denote the contribution weights of different views. In this study, these weights are empirically set to 0.58, 0.27, and 0.15, respectively, to balance spectral structure and temporal dynamics.

Through this design, the proposed representation simultaneously captures spectral structure, noise-robust characteristics, and temporal dynamics, providing a more informative and discriminative input for subsequent deep acoustic modeling.

#### 2.4.3. Segment-Level Acoustic Modeling with CNN-Conformer-MIL

**Figure 3:**
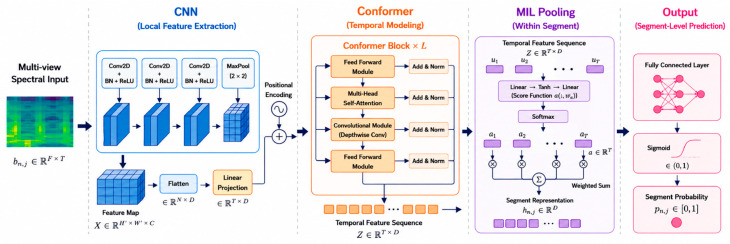
Architecture of the proposed CNN-Conformer-MIL model for segment-level acoustic modeling.

Based on the multi-view spectral representation constructed in Section 2.4.2, a CNN-Conformer network is employed to perform segment-level prediction, followed by a temporal attention pooling mechanism. The overall framework of the proposed segment-level modeling architecture is illustrated in Figure 3.

For each bowel sound segment $b_{n,j}$, the input feature $S_{n,j}$ corresponds to the fused multi-view spectral representation $S_{m,v}$, which is resized to a fixed resolution of 32 × 32 before being fed into the network. The model learns a mapping from the spectral representation to the probability of the segment:

(20) $$p_{n,j}=f_{\theta }(S_{n,j})$$

where $f_{\theta }(\cdot )$ denotes the deep acoustic model parameterized by θ.

The input multi-view spectral patch is first processed by a convolutional stem to extract local time-frequency patterns. Specifically, three convolutional blocks are used with channel dimensions of 32, 48, and 64, respectively. Each block consists of a 3×3 convolution, batch normalization, and SiLU activation, while two 2×2 max-pooling operations are applied to progressively reduce the spatial resolution. Through this process, local spectral structures such as short-duration acoustic events and local frequency transitions can be effectively captured.

The resulting feature map is then rearranged into a temporal token sequence and projected into a compact embedding space for contextual modeling. In the implemented architecture, the feature sequence is mapped to a 96-dimensional representation and subsequently processed by two stacked Conformer blocks [26]. Each Conformer block integrates feed-forward transformation, multi-head self-attention [27], and convolutional modeling, enabling the network to jointly capture global temporal dependencies and local contextual continuity within each bowel sound segment. The self-attention mechanism is defined as

(21) $$Attn(Q,K,V)=softmax(\frac{QK^{T}}{\sqrt{d}})V$$

where *Q*, *K*, and *V* denote the query, key, and value matrices, respectively, and d is the feature dimension. In this study, the Conformer module uses 4 attention heads, a model dimension of 96, a dropout rate of 0.1, and a depthwise convolution kernel size of 9.

To summarize the temporal representations within each segment, an attention-based temporal pooling mechanism, formulated in an MIL-style manner, is introduced [28]. Let $u_{t}$ denote the feature at time step t. The segment-level representation is computed as

(22) $$h_{n,j}=\sum_{t=1}^{T}\alpha_{t}u_{t},\;\;\alpha_{t}=\frac{\exp(a(u_{t}))}{\sum_{k=1}^{T}\exp(a(u_{k}))}$$

where $\alpha_{t}$ denotes the attention weight assigned to the t-th temporal feature, reflecting its relative importance within the temporal structure of the segment. The attention score function a(⋅) is implemented by a lightweight projection network followed by Tanh activation and softmax normalization, allowing the model to focus on more informative acoustic patterns while suppressing less relevant temporal regions. It is important to note that this mechanism operates at the temporal level within each segment, rather than across multiple segments at the patient level.

Finally, the aggregated segment representation is passed through a fully connected layer followed by a sigmoid activation to obtain the prediction probability:

(23) $$p_{n,j}=\sigma ({\mathrm{Wh}}_{n,j}+b)$$

Through this deep acoustic modeling process, the proposed multi-view spectral representation is transformed into discriminative segment-level predictions, which subsequently serve as the basis for downstream patient-level aggregation and classification.

#### 2.4.4. Patient-Level Decision Modeling

Based on the segment-level predictions obtained in Section 2.4.3, patient-level modeling is performed to infer the final stool status. For each patient n, a set of segment-level prediction probabilities ${\left\{p_{n,j}\right\}}_{j=1}^{m_{n}}$ is available, where $m_{n}$ denotes the number of segments. Instead of relying on a single aggregated score, the proposed method models the distribution of segment-level predictions to obtain a more robust and reliable patient-level decision.

Specifically, a set of statistical descriptors is constructed to characterize the prediction distribution from multiple perspectives, including global tendency, extremal responses, and dispersion properties. These descriptors include the mean, standard deviation, maximum, minimum, and percentile-based statistics (25th, 50th, and 75th percentiles). Together, they form a compact feature vector $z_{n}\in {\mathbb{R}}^{d}$, where d=8 in this study. In addition, an uncertainty measure is incorporated to capture prediction consistency across segments, such as variance or dispersion, enabling unreliable or noisy segments to be implicitly down-weighted during modeling.

Based on these statistical components, an initial patient-level prediction can be formulated through weighted aggregation:

(24) $$p_{n}=\sum_{k=1}^{d}\beta_{k}\cdot \;\Phi_{k}({\left\{p_{n,j}\right\}}_{j=1}^{M_{n}})$$

where $\Phi_{k}(\cdot )$ denotes statistical operators applied to the prediction set and $\beta_{k}$ represents their corresponding weights. This formulation integrates both global tendency and local distributional characteristics of segment-level outputs.

To further enhance prediction reliability, a patient-level calibration strategy is introduced as a key component of the framework. Instead of directly using the aggregated score, the constructed statistical descriptors are treated as features and mapped to the final prediction through a learnable function. The calibrated prediction is defined as

(25) $${\tilde{p}}_{n}=\sigma (w^{⊤}z_{n}+b)$$

where σ(·) is the sigmoid function and $w\in {\mathbb{R}}^{d},b\in \mathbb{R}$ denote the model parameters. In practice, this mapping is implemented as a lightweight linear classifier trained using binary cross-entropy loss. This design enables the model to learn an adaptive decision boundary over the distribution of segment-level predictions.

This calibration process can be interpreted as learning a mapping from the distribution of segment-level outputs to the final patient-level decision. By jointly modeling global tendency, extremal responses, and prediction consistency, the proposed approach effectively corrects biased or unstable aggregation results, transforming the aggregation process from a fixed heuristic into a learnable decision function.

## 3. Experimental Data and Setup

### 3.1. Data Acquisition and Annotation

The bowel sound data used in this study were collected from the Sichuan Provincial Second Hospital of Traditional Chinese Medicine. A total of 92 subjects were enrolled, each contributing one continuous abdominal audio recording, resulting in 92 independent patient-level samples. Each recording has a duration of approximately 3 min, ensuring sufficient temporal coverage for subsequent analysis while maintaining patient-level independence.

Data acquisition was performed using a smart digital stethoscope developed by Jingchuang Gaoweidu Technology Co., Ltd. (Chengdu, China). The device is characterized by its compact size, high-fidelity signal acquisition, and low power consumption, making it suitable for continuous abdominal acoustic signal collection in real-world clinical environments.

During bowel sound acquisition, all participants were recorded in a quiet resting supine position, with auscultation sites standardized at two locations 5 cm lateral to the umbilicus on both sides. To minimize the influence of short-term physiological and behavioral factors, participants were asked to remain at rest and avoid vigorous physical activity before recording. It should be noted that factors such as blood pressure, heart rate, recent activity, food intake, and intestinal gas may influence bowel sound characteristics. However, the present study focused on evaluating the feasibility of the proposed two-stage bowel sound detection and patient-level classification framework, and these factors were not explicitly modeled as independent covariates. Future studies may further incorporate these physiological and behavioral variables to improve model robustness and clinical generalizability.

To ensure data reliability and annotation consistency, a structured annotation protocol was adopted. Specifically, salient bowel sound events were manually annotated by professional physicians from the Sichuan Provincial Second Hospital of Traditional Chinese Medicine. The annotations were subsequently reviewed and refined through a consensus process to resolve inconsistencies and improve labeling accuracy.

For classification tasks, labels were assigned based on the BSFS, which serves as a clinically validated reference for assessing bowel conditions. In the binary classification setting, samples were categorized into constipation (types 1–3) and non-constipation (types 4–7). The detailed label definitions are summarized in Table 1.

All data were anonymized prior to analysis to protect patient privacy. This study was approved by the Medical Ethics Committee of Sichuan Provincial Second Hospital of Traditional Chinese Medicine (Approval No. 2024zx-10, approved on 12 November 2024). The study was conducted in accordance with the Declaration of Helsinki and relevant ethical guidelines for human-subject research. Written informed consent was obtained from all participants prior to data collection.

### 3.2. Data Splitting Strategy

To ensure a fair and reliable evaluation, data splitting was performed at the patient level for both tasks, while adopting task-specific strategies to account for differences in data scale and modeling objectives.

(1) Task 1: Salient Bowel Sound Detection

For salient bowel sound detection, each subject’s continuous recording was first segmented into candidate acoustic segments using the sliding-window strategy described in Section 2.3.1. These segments were then labeled based on their temporal overlap with annotated bowel sound events.

To avoid information leakage, data splitting was conducted at the patient level. Specifically, the dataset was divided into training, validation, and test sets using a ratio of 6:2:2. All segments belonging to the same subject were assigned to the same subset, ensuring that no segment from a given subject appeared in multiple sets.

This strategy allows the detection model to be evaluated under realistic conditions, where it must generalize to unseen subjects rather than memorizing subject-specific acoustic patterns.

(2) Task 2: Patient-Level Constipation Classification

For patient-level constipation classification, segment construction follows the event-driven strategy described in Section 2.4.1. Specifically, salient bowel sound events identified by the detection stage are used to construct informative segments for each subject. These segments are then grouped at the patient level to form the input for classification.

Given the relatively limited number of subjects (92 in total), a five-fold cross-validation (five-fold CV) strategy was adopted to ensure robust and unbiased performance estimation. The dataset was partitioned into five mutually exclusive folds at the patient level, where in each iteration, three folds were used for training, one fold for validation, and one fold for testing.

Within each cross-validation iteration, the training, validation, and test folds were kept strictly separated at the patient level. The validation fold was used for model selection, validation-based threshold determination, and patient-level calibration, whereas the test fold was held out until the final evaluation of that iteration. Importantly, no information from the test fold was used for threshold selection, feature selection, patient-level calibration, early stopping, hyperparameter tuning, or any other model-tuning procedure.

To maintain balanced class distributions across folds, stratified sampling was applied based on patient-level labels. This ensures that each fold preserves a similar proportion of different stool categories.

### 3.3. Training and Implementation Details

All signal preprocessing, feature extraction, model training, and statistical analyses were implemented in Python v3.13.9. The deep learning models were implemented using PyTorch v2.7.0. The main Python packages included NumPy v2.1.3, SciPy v1.16.3, scikit-learn v1.7.2, librosa v0.11.0, PyWavelets v1.8.0, pandas v3.0.1, and Matplotlib v3.10.6. The development environment used was PyCharm v2025.2.1.

#### 3.3.1. Training Details for Salient Bowel Sound Detection

For salient bowel sound detection, the preprocessing and candidate segment construction procedures followed the strategy described in Section 2.3.1. Specifically, continuous abdominal recordings were resampled to 4000 Hz and band-pass filtered within 80–1800 Hz, and candidate segments were generated using a sliding window of 0.5 s with a hop size of 0.1 s. Segment labels were assigned according to their temporal overlap with annotated bowel sound events.

To improve data quality and address class imbalance, an event-centered sampling strategy was adopted, together with a two-stage negative sampling scheme. In addition, jittered sampling around event centers was applied to enhance robustness. Each candidate segment was represented using fused handcrafted features and log-Mel-based time-frequency features, which were further refined into a compact 60-dimensional representation before being fed into the CNN-MAMBA detection model.

The model was trained using the Adam optimizer with a learning rate of 5 × 10^−4^, batch size of 32, and weight decay of 1 × 10^−4^ for up to 40 epochs. Early stopping with a patience of 10 epochs was applied to prevent overfitting. Focal loss was employed to mitigate the impact of class imbalance, and model selection was based on validation AUC.

These training settings were designed to improve robustness under sparse-event and imbalanced-data conditions, which are characteristic of salient bowel sound detection in continuous abdominal recordings.

#### 3.3.2. Training Details for Patient-Level Constipation Classification

For patient-level classification, bowel sound segments were converted into log-Mel spectrograms with 64 Mel bands. The FFT size was adaptively selected according to the segment length within a range of 64 to 512, and the hop length was set to one quarter of the FFT size. All spectrograms were normalized and resized to 32 × 32 before being used as model inputs.

The CNN-Conformer-based acoustic model was trained using the AdamW optimizer. The initial learning rate was set to 7 × 10^−4^, and the weight decay was set to 1 × 10^−4^. The batch size was 16, and the maximum number of training epochs was 32. Early stopping was applied with a patience of 7 epochs. Gradient clipping was performed with a maximum norm of 2.0.

To address class imbalance, weighted random sampling and dynamically calculated positive class weighting were used during training. The loss function was binary cross-entropy with logits, incorporating class weights. A ReduceLROnPlateau scheduler was used to adjust the learning rate based on validation performance.

Lightweight data augmentation was applied only to the training set, including Gaussian noise injection, time masking, frequency masking, temporal shifting, and amplitude scaling.

During inference, segment-level prediction probabilities were aggregated into patient-level predictions using the MIL-based aggregation strategy. The classification threshold was selected on the validation set mainly according to the F1-score, with recall used as an additional reference.

Model performance was evaluated using patient-level five-fold cross-validation, and the results were averaged across all folds.

### 3.4. Evaluation Metrics

To ensure a clear and consistent evaluation protocol, three types of metrics are defined in this study: model selection metrics, threshold selection metrics, and final reported metrics.

(1) Model Selection Metrics

For Task 1 (salient bowel sound detection), model selection is performed based on the validation ROC-AUC, as it reflects the overall discriminative capability under class imbalance conditions.

For Task 2 (patient-level constipation classification), model selection is based on the validation F1-score, which provides a balanced assessment between precision and recall under limited-sample settings.

(2) Threshold Selection Metrics

For both tasks, the classification threshold is determined on the validation set.

For Task 1, the threshold is selected by maximizing the F1-score on the validation set.

For Task 2, the threshold is determined by jointly considering multiple balance-related metrics, including F1-score and recall, to achieve a stable trade-off between sensitivity and precision. The selected threshold is then fixed and applied to the corresponding test set or validation fold.

(3) Final Reported Metrics

For Task 1, performance is evaluated at the segment level using Accuracy, Precision, Recall, F1-score, and ROC-AUC on the held-out test set.

For Task 2, performance is evaluated at the patient level using Accuracy, Precision, Recall, and F1-score, with results reported as the mean and standard deviation across five-fold cross-validation.

ROC-AUC is reported as a supplementary metric where applicable and is consistently included when presented in comparative tables.

## 4. Results

### 4.1. Task 1: Salient Bowel Sound Detection

Salient bowel sound detection serves as the foundation for the subsequent patient-level classification task. This task is inherently challenging due to the short duration, sparsity, and non-stationary nature of bowel sound events, which are often easily affected by background interference.

Therefore, in addition to reporting overall detection performance, further analyses are conducted from multiple perspectives, including threshold selection, feature representation, and model robustness, to provide a more comprehensive understanding of model behavior.

#### 4.1.1. Overall Classification Performance

In this study, we evaluated the performance of segment-level salient bowel sound detection under a patient-level data split to avoid subject leakage between training and evaluation. The dataset consisted of 92 subjects and 24,602 audio segments in total. Due to the sparse and short-duration nature of salient bowel sound events, the dataset exhibited a clear class imbalance, with a positive-to-negative ratio of approximately 1:4.26.

Table 2 summarizes the overall performance of different models on the test set. Conventional deep learning models, such as CNN and BiLSTM, achieve baseline performance but show a trade-off between precision and recall. Sequence modeling approaches, including Transformer and TCN, further improve the results, achieving F1-scores of 0.73–0.74, indicating their effectiveness in capturing temporal dependencies.

In contrast, the proposed CNN-MAMBA achieves the best performance across all evaluation metrics, with an accuracy of 0.87, a precision of 0.79, a recall of 0.77, an F1-score of 0.78, and an ROC-AUC of 0.93. These results demonstrate that integrating convolutional feature extraction with advanced sequence modeling, together with feature fusion and dynamic thresholding strategies, significantly enhances classification performance.

**Figure 4:**
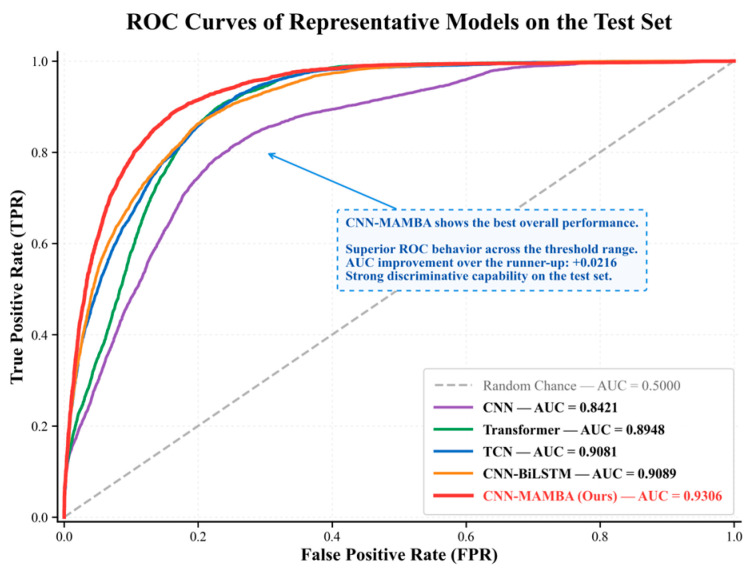
ROC curves of representative models on the test set.

To further illustrate the discriminative capability of different models, Figure 4 presents the ROC curves on the test set. It can be observed that CNN-MAMBA consistently achieves the highest curve across all thresholds, clearly outperforming the baseline models.

Overall, the proposed method demonstrates strong effectiveness for prominent bowel sound classification and provides a solid foundation for subsequent analyses on thresholding strategies and feature fusion mechanisms.

#### 4.1.2. Impact of Dynamic Thresholding Strategy

To further investigate the impact of threshold selection on classification performance, we compare static and dynamic thresholding strategies on the test set. Specifically, a fixed threshold of 0.50 is adopted as the conventional baseline, while a dynamic threshold is determined based on validation optimization and applied to the test set.

Table 3 presents the quantitative comparison between the two strategies. Although the static threshold achieves slightly higher precision (0.82), it suffers from relatively low recall (0.68), indicating that a considerable number of prominent bowel sound segments are missed. In contrast, the dynamic threshold (0.40) significantly improves recall (0.77), while maintaining a competitive precision (0.79), leading to a higher F1-score (0.78 vs. 0.74). Notably, the ROC-AUC remains consistent (0.93), suggesting that the improvement mainly stems from a better operating point rather than changes in model discrimination capability.

Overall, these results indicate that appropriate threshold selection plays an important role in handling class imbalance in salient bowel sound detection. By adjusting the decision boundary, the proposed method improves sensitivity to relevant bowel sound events while maintaining stable overall performance.

#### 4.1.3. Ablation and Comparative Analysis

To comprehensively evaluate the effectiveness of the proposed method, we conduct a unified ablation and comparative analysis from two perspectives: feature representation and model architecture.

#### 4.1.4. Impact of Feature Representation on Model Performance

To investigate the impact of different feature representations, we conducted an ablation study comparing learned deep features, handcrafted acoustic features, and their combined representation used as model input. The results are summarized in Table 4.

As shown in Table 4, different feature representations lead to distinct performance characteristics in salient bowel sound detection. The CNN-MAMBA model without handcrafted feature input achieves the highest precision of 0.86, but its recall is only 0.46, resulting in an F1-score of 0.60. This indicates that the model is relatively conservative in identifying positive bowel sound events. Although the predicted positive samples are more likely to be correct, many true bowel sound events are missed.

When handcrafted acoustic features are introduced, the recall increases substantially from 0.46 to 0.82, while the precision decreases from 0.86 to 0.53. This suggests that handcrafted features help the model capture a broader range of bowel sound events, including weak or atypical acoustic patterns. However, the increased sensitivity also introduces more false positives, leading to a lower accuracy of 0.73.

After feature refinement is applied to the handcrafted feature representation, the model achieves the best overall performance, with an accuracy of 0.87, a precision of 0.79, a recall of 0.77, an F1-score of 0.78, and an ROC-AUC of 0.93. Compared with using handcrafted features alone, feature refinement improves the balance between precision and recall and increases the F1-score from 0.64 to 0.78. This indicates that feature refinement effectively reduces redundancy and retains more discriminative acoustic information.

Overall, these results show that handcrafted acoustic features can improve the sensitivity of bowel sound detection, while feature refinement further enhances feature compactness and discriminative ability.

#### 4.1.5. Comparative Analysis of Model Architectures

In addition to the analysis of feature representations, we further evaluate the impact of different model architectures by comparing CNN, MAMBA, and the proposed CNN-MAMBA under the same experimental settings. The results are summarized in Table 5.

As shown in Table 5, different model architectures exhibit distinct characteristics in modeling bowel sound signals. The CNN model achieves relatively stable performance, with an accuracy of 0.79 and F1-score of 0.66, benefiting from its strong ability to capture local time-frequency patterns. However, its recall (0.69) remains limited, indicating insufficient sensitivity to certain transient or low-intensity bowel sound events, which are common in real-world recordings.

In contrast, the MAMBA model attains higher accuracy (0.82) and precision (0.81), suggesting improved discriminative capability in distinguishing bowel sounds from background noise. This reflects its advantage in sequence modeling and its ability to capture global temporal dependencies. However, its recall drops to 0.53, indicating that it may miss a considerable number of positive bowel sound segments, possibly due to over-smoothing of local acoustic variations.

The proposed CNN-MAMBA model achieves the best overall performance across all evaluation metrics, with an accuracy of 0.87, precision of 0.79, recall of 0.77, F1-score of 0.78, and ROC-AUC of 0.93. Compared with the individual models, it achieves a more balanced trade-off between precision and recall.

These results indicate that CNN and MAMBA provide complementary modeling capabilities for bowel sound analysis. While CNN focuses on capturing fine-grained local acoustic patterns, MAMBA emphasizes long-range temporal dependencies. By integrating both components, the proposed hybrid architecture captures both local and global characteristics of bowel sound signals and showed improved detection performance within the current dataset.

#### 4.1.6. Robustness and Prediction Distribution Analysis

**Figure 5:**
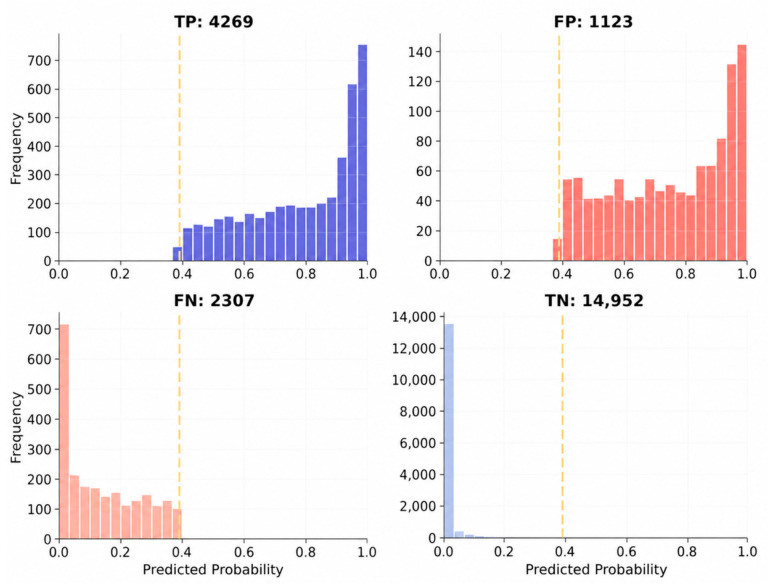
Prediction score distributions of TP, FP, FN, and TN samples on the test set.

To further evaluate the reliability and stability of the proposed model, we analyze the distribution of prediction scores on the test set, as shown in Figure 5.

Figure 5 illustrates the predicted probability distributions of true positives (TPs), false positives (FPs), false negatives (FNs), and true negatives (TNs) under the operating threshold of 0.40. It can be observed that most TP samples are distributed above the threshold, with a large proportion concentrated in the high-confidence region close to 1.0. This indicates that the model assigns strong confidence to correctly identified prominent bowel sound events.

In contrast, TN samples are highly concentrated near zero, far below the decision threshold, which demonstrates that the model can effectively suppress background segments and maintain stable negative recognition. This clear separation between TPs and TNs explains the strong overall discriminative performance of the proposed framework.

For misclassified samples, the FP distribution is mainly located above the threshold and extends toward high-confidence regions, suggesting that some background segments share acoustic patterns similar to true prominent bowel sounds. Meanwhile, FN samples are mostly distributed below the threshold, with many concentrated in the low-probability region, indicating that weak, ambiguous, or less typical bowel sound events are more likely to be missed.

Overall, the prediction score distributions show that the selected threshold of 0.40 provides an effective operating point for separating most positive and negative samples. At the same time, the remaining FP and FN cases reflect the intrinsic difficulty of borderline samples, indicating that practical robustness should be interpreted cautiously and further examined in larger external datasets.

Overall, the proposed CNN-MAMBA framework showed favorable segment-level detection performance across the evaluated metrics, but these findings should be considered dataset-specific evidence requiring further validation.

### 4.2. Task 2: Patient-Level Constipation Classification

Patient-level constipation classification builds upon bowel sound analysis and aims to determine whether a subject is constipated based on acoustic signals. This task is challenging due to strong inter-subject variability and the need to aggregate sparse and noisy segment-level bowel sound events into reliable patient-level predictions. Therefore, beyond reporting overall performance, it is necessary to examine the impact of feature representation and patient-level decision strategies. To this end, four experimental configurations are designed by combining different spectral representations with the presence or absence of patient-level calibration, enabling a systematic evaluation of their contributions to classification performance.

#### 4.2.1. Overall Classification Performance

In this task, we evaluate the performance of representative models for patient-level constipation classification based on bowel sound signals. The results are summarized in Table 6.

As shown in Table 6, conventional deep learning models such as CNN and CNN-BiLSTM achieve moderate performance, with F1-scores around 0.69, indicating their limited capacity to capture complex acoustic variations in bowel sound segments. Although the CNN-Conformer model enhances temporal modeling through attention mechanisms, its performance remains relatively constrained, suggesting that sequential modeling alone is insufficient to handle the noisy and highly variable nature of bowel sound signals.

More advanced architectures further improve performance. The Hybrid-Hierarchical-MIL model achieves an F1-score of 0.724, demonstrating the effectiveness of structured aggregation strategies, while the Audio-Mamba model yields competitive results, highlighting the importance of modeling long-range dependencies.

The proposed CNN-Conformer-MIL model achieves the highest mean F1-score among the compared patient-level models, with an accuracy of 0.665, a precision of 0.758, a recall of 0.760, and an F1-score of 0.755. However, the absolute improvement over several alternative models is modest, and the standard deviations indicate non-negligible variability across folds; therefore, the patient-level findings should be interpreted as preliminary feasibility evidence rather than definitive model superiority. Compared with the CNN-Conformer model, this improvement suggests that incorporating attention-based MIL pooling enables more effective emphasis on informative temporal components while mitigating the influence of noisy or less relevant segments.

Overall, the results indicate that improving segment-level representations and adopting more effective aggregation mechanisms are crucial for enhancing patient-level classification performance.

#### 4.2.2. Effect of Multi-View Spectral Representation

In this section, we evaluate the contribution of the proposed multi-view spectral representation to patient-level constipation classification. The proposed representation integrates Log-Mel spectrogram, PCEN representation, and temporal delta features, which provide complementary information regarding spectral energy distribution, weak-event enhancement, and transient temporal variation.

To assess its effectiveness, we compared the CNN-Conformer-MIL model using Log-Mel input and multi-view input under the same patient-level calibration setting. The results are summarized in Table 7.

As shown in Table 7, the multi-view representation improves accuracy from 0.6463 to 0.6651, precision from 0.7267 to 0.7576, and F1-score from 0.7500 to 0.7545. These improvements indicate that integrating complementary spectral views enhances the discriminative capacity of the segment-level representation. Although recall slightly decreases from 0.7793 to 0.7596, it remains comparable to the Log-Mel baseline, suggesting that the proposed representation improves overall prediction quality without substantially compromising sensitivity.

Overall, the results suggest that the proposed multi-view spectral representation may be beneficial for patient-level constipation classification, although the magnitude of improvement was small. By combining spectral structure, noise-robust enhancement, and temporal dynamic information, it provides more comprehensive acoustic features for the CNN-Conformer-MIL model and contributes to more stable patient-level prediction.

#### 4.2.3. Comparative Analysis of Patient-Level Calibration

To analyze the effect of patient-level calibration, we compare the performance of the CNN-Conformer-MIL model before and after calibration under the multi-view spectral representation, as summarized in Table 8.

As shown in Table 8, introducing patient-level calibration leads to consistent improvements across evaluation metrics. The F1-score increases from 0.7425 to 0.7545. Meanwhile, accuracy rises from 0.6457 to 0.6651, precision improves from 0.7500 to 0.7576, and recall increases from 0.7429 to 0.7596. These results suggest that patient-level calibration helps refine the final decision by incorporating the distribution of segment-level predictions.

Overall, the results indicate that patient-level calibration remains beneficial under the multi-view spectral representation, contributing to improved stability and consistency of patient-level classification.

## 5. Discussion and Conclusions

All BSFS labels were annotated by clinical physicians and aligned with bowel sound recordings to ensure correspondence between acoustic signals and labeled conditions. Notably, BSFS types 3 and 4 are adjacent on the stool form scale but were assigned to different binary categories, which may introduce boundary ambiguity and partly explain the moderate patient-level classification accuracy.

We proposed a two-stage framework for bowel sound-based gastrointestinal assessment in elderly subjects, combining salient bowel sound detection (CNN-MAMBA) and patient-level classification (CNN-Conformer-MIL with multi-view features). Extracting informative bowel sound events is crucial because bowel sounds are sparse, transient, and easily affected by noise. Given the moderate patient-level performance, these results should be interpreted as preliminary feasibility evidence rather than definitive clinical diagnosis.

Physiologically, bowel sounds relate to intestinal motility, gas, and fluid movement, while stool form reflects intestinal transit. Therefore, associations between bowel sounds and BSFS-based constipation status are plausible but indirect.

This framework provides a feasibility-oriented approach for auxiliary bowel health screening, home monitoring, or follow-up assessment. Limitations include the small single-center dataset, simplified BSFS grouping, and short recording duration. Future work should involve larger multicenter datasets, longer recordings, finer-grained BSFS modeling, external validation, and integration of clinical data. Overall, bowel sound analysis shows potential for low-burden gastrointestinal health monitoring but is not a replacement for clinician-led diagnosis.

## Figures and Tables

**Table 1:** Definition of bowel condition based on the BSFS.

BSFS Type	Description	Clinical Interpretation	Category (Binary)
Type 1	Separate hard lumps	Severe constipation	Constipation
Type 2	Lumpy and sausage-like	Moderate constipation	Constipation
Type 3	Sausage with cracks on the surface	Mild constipation	Constipation
Type 4	Smooth and soft sausage-like	Normal	Non-constipation
Type 5	Soft blobs with clear-cut edges	Slightly loose	Non-constipation
Type 6	Fluffy pieces with ragged edges	Mild diarrhea tendency	Non-constipation
Type 7	Watery, no solid pieces	Severe diarrhea	Non-constipation

**Table 2:** Overall performance comparison of representative models on the test set.

Model	Accuracy	Precision	Recall	F1-Score	ROC-AUC	Threshold
CNN	0.79	0.63	0.69	0.66	0.84	0.30
BiLSTM [29]	0.81	0.71	0.63	0.67	0.85	0.50
CNN-BiLSTM	0.84	0.72	0.74	0.73	0.90	0.25
Transformer	0.82	0.69	0.85	0.74	0.90	0.41
TCN [30]	0.84	0.77	0.69	0.73	0.90	0.05
RetNet [31]	0.81	0.73	0.61	0.67	0.85	0.39
Conformer	0.54	0.50	0.82	0.62	0.61	0.50
Hyena [32]	0.81	0.72	0.63	0.67	0.88	0.50
SE-Attention-TCN [33]	0.80	0.65	0.78	0.71	0.89	0.47
CNN-MAMBA (Ours)	0.87	0.79	0.77	0.78	0.93	0.40

**Table 3:** Performance comparison between static and dynamic thresholding strategies using CNN-MAMBA.

Type	Accuracy	Precision	Recall	F1-Score	ROC-AUC	Threshold
Static	0.86	0.82	0.68	0.74	0.93	0.50
Dynamic	0.87	0.79	0.77	0.78	0.93	0.40

**Table 4:** Ablation study of different feature representations for salient bowel sound detection.

Model (Feature)	Accuracy	Precision	Recall	F1-Score	ROC-AUC
CNN-MAMBA	0.82	0.86	0.46	0.60	0.84
CNN-MAMBA (Handcrafted features)	0.73	0.53	0.82	0.64	0.86
CNN-MAMBA (Handcrafted features with feature refinement)	0.87	0.79	0.77	0.78	0.93

**Table 5:** Comparison of different model architectures for salient bowel sound detection.

Model	Accuracy	Precision	Recall	F1-Score	ROC-AUC
CNN	0.79	0.63	0.69	0.66	0.84
MAMBA	0.82	0.81	0.53	0.64	0.85
CNN-MAMBA	0.87	0.79	0.77	0.78	0.93

**Table 6:** Comparison of representative models for patient-level constipation classification. The results of the proposed model are highlighted in bold.

Model	Accuracy	Precision	Recall	F1-Score
CNN	0.611 ± 0.045	0.764 ± 0.055	0.648 ± 0.136	0.688 ± 0.068
CNN-BiLSTM	0.603 ± 0.145	0.722 ± 0.126	0.687 ± 0.190	0.694 ± 0.138
CNN-Conformer	0.565 ± 0.092	0.734 ± 0.070	0.603 ± 0.179	0.644 ± 0.100
Hybrid-Hierarchical-MIL	0.613 ± 0.066	0.710 ± 0.048	0.766 ± 0.170	0.724 ± 0.070
Audio-Mamba	0.577 ± 0.088	0.736 ± 0.102	0.642 ± 0.073	0.678 ± 0.046
**CNN-Conformer-MIL (Ours)**	**0.665 ± 0.074**	**0.758 ± 0.047**	**0.760 ± 0.106**	**0.755 ± 0.062**

**Table 7:** Performance of the CNN-Conformer-MIL model before and after multi-view spectral representation under patient-level calibration. The results of the proposed model are highlighted in bold.

Feature Setting	Accuracy	Precision	Recall	F1-Score
Log-Mel (Before)	0.6463 ± 0.0686	0.7267 ± 0.0380	0.7793 ± 0.0948	0.7500 ± 0.0581
**Multi-View** **(After)**	**0.6651 ± 0.0743**	**0.7576 ± 0.0474**	**0.7596 ± 0.1060**	**0.7545 ± 0.0622**

**Table 8:** Effect of patient-level calibration on model performance under the multi-view spectral representation.

Calibration	Accuracy	Precision	Recall	F1-Score
Before	0.6457 ± 0.0557	0.7500 ± 0.0511	0.7429 ± 0.0708	0.7425 ± 0.0358
After	0.6651 ± 0.0743	0.7576 ± 0.0474	0.7596 ± 0.1060	0.7545 ± 0.0622

## Data Availability

The datasets generated and analyzed during the current study are not publicly available due to privacy and ethical restrictions involving human participants. De-identified data may be available from the corresponding author upon reasonable request and with approval from the relevant ethics committee and institution.
